# Three episodes of recurrent Takotsubo syndrome pre- and post-heart transplantation: a case report

**DOI:** 10.1093/ehjcr/ytaf213

**Published:** 2025-04-28

**Authors:** Kaori Yasumura, Fusako Sera, Yasuhiro Akazawa, Tomohito Ohtani, Yasushi Sakata

**Affiliations:** Department of Cardiovascular Medicine, The University of Osaka Graduate School of Medicine, 2-15 Yamadaoka, Suita 565-0871, Japan; Department of Cardiovascular Medicine, The University of Osaka Graduate School of Medicine, 2-15 Yamadaoka, Suita 565-0871, Japan; Department of Cardiovascular Medicine, The University of Osaka Graduate School of Medicine, 2-15 Yamadaoka, Suita 565-0871, Japan; Department of Cardiovascular Medicine, The University of Osaka Graduate School of Medicine, 2-15 Yamadaoka, Suita 565-0871, Japan; Department of Cardiovascular Medicine, The University of Osaka Graduate School of Medicine, 2-15 Yamadaoka, Suita 565-0871, Japan

**Keywords:** Case report, Cardiac sympathetic hyperactivity, Heart transplantation, Iodine-123 meta iodobenzylg uanidine (^123^I-MIBG) scintigraphy, Recurrence, Sympathetic reinnervation, Takotsubo syndrome

## Abstract

**Background:**

Cardiac sympathetic hyperactivity may be implicated in the pathogenesis of Takotsubo syndrome (TTS). With complete denervation of a transplanted heart, the recipient heart is less susceptible to sympathetic hyperactivity. We report a rare case of recurrent TTS in a heart transplant recipient from a donor with TTS. Iodine-123 meta-iodobenzylguanidine (^123^I-MIBG) scintigraphy results for evaluating sympathetic activity are presented.

**Case Summary:**

A 46-year-old woman underwent heart transplantation for dilated phase of hypertrophic cardiomyopathy following recurrent cerebral haemorrhage complications after left ventricular assist device therapy 2 years prior. The donor heart exhibited a transient mildly reduced left ventricular ejection fraction suggestive of TTS. Four years post-transplantation, she was admitted with difficulty breathing, and echocardiography showed decreased biventricular apical wall motion. During her treatment course, wall motion improved spontaneously, and giant negative T waves were observed on electrocardiography (ECG). Coronary computed tomography and endomyocardial biopsy findings were normal, and a diagnosis of TTS was made. Approximately 5 years post-transplantation, she was readmitted with difficulty breathing, and echocardiography showed akinesis of the mid-to-apical biventricular wall. Wall motion normalized within a few days, and ECG showed typical giant negative T waves, consistent with TTS. ^123^I-MIBG scintigraphy taken prior to discharge and 6 months later showed minimal myocardial uptake in the basal anterior wall, indicating insufficient sympathetic reinnervation.

**Discussion:**

Recurrent TTS in a denervated heart suggests the possibility of other underlying mechanisms besides cardiac sympathetic hyperactivity. This case highlights potential TTS development in transplant recipients from donors with TTS, underlining the need for close monitoring.

**Learning points**

- Takotsubo syndrome (TTS) can occur in patients with insufficient sympathetic reinnervation following heart transplantation independently of cardiac sympathetic hyperactivity.
- Recipients transplanted from donors with TTS require close monitoring owing to the risk of TTS recurrence.

## Introduction

Takotsubo syndrome (TTS) is an acute, reversible condition characterized as systolic ventricular dysfunction. Several hypotheses concerning its pathogenesis have been proposed, among which cardiac sympathetic hyperactivity is prominent.^1^

Meta-iodobenzylguanidine, a norepinephrine analogue, is commonly utilized in iodine-123 meta-iodobenzylguanidine (^123^I-MIBG) scintigraphy to assess cardiac sympathetic nerve function. Early-phase images primarily reflect the number of functioning nerve terminals, whereas washout rates correlate with sympathetic activity.^2^ In patients with TTS, decreased uptake, particularly in delayed images, indicates significantly increased washout, suggesting cardiac sympathetic hyperactivity.^3^ Additionally, ^123^I-MIBG scintigraphy is used to evaluate sympathetic reinnervation in transplanted hearts, which are completely denervated at the time of transplantation, resulting in defective ^123^I-MIBG uptake.^4^

We report a rare case of two recurrent TTS episodes after heart transplantation from a donor with TTS. To investigate sympathetic reinnervation involvement in this context, ^123^I-MIBG scintigraphy was performed in the acute and chronic phases.

## Summary figure

**Figure:**
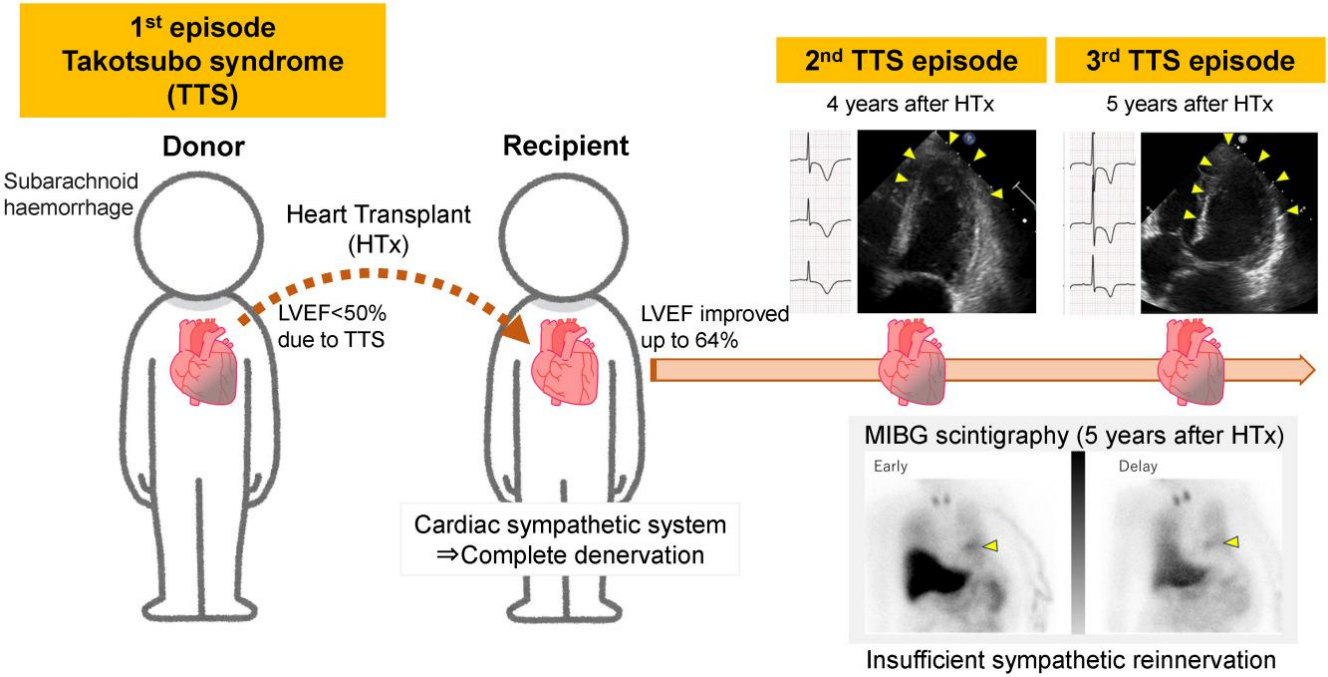


## Case presentation

**Figure 1:**
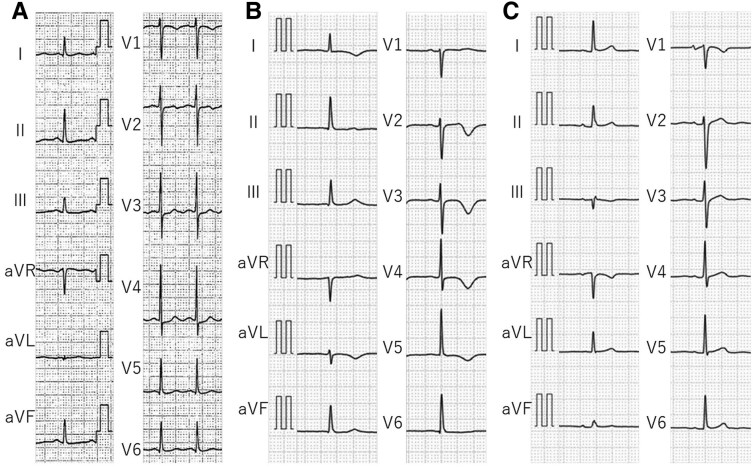
Electrocardiography during the first takotsubo syndrome episode. Electrocardiography of the donor 2days prior to organ donation (*A*). Electrocardiography of the recipient immediately (*B*) and 3 months (*C*) post-transplantation.

A 46-year-old woman underwent heart transplantation at our institution. She was diagnosed with dilated phase of hypertrophic cardiomyopathy 6-years prior, which progressed to end-stage heart failure requiring left ventricular assist device (LVAD) therapy 2-years prior. However, she developed recurrent cerebral haemorrhages owing to LVAD complications, necessitating heart transplantation from a marginal donor with a mildly reduced left ventricular ejection fraction (LVEF). The donor became brain dead owing to a subarachnoid haemorrhage 4 days before donation, with mild ST depression observed on electrocardiography (ECG) 2 days prior (*Figure 1A*). A pre-transplant echocardiography showed reduced LV contractility, LVEF <50%, and moderate hypokinesis of the mid-to-apical LV wall. Immediately post-transplantation, the patient exhibited negative T-waves on ECG (*Figure 1B*), which improved 3 months later (*Figure 1C*); moreover, LV wall motion normalized (LVEF 64%; Supplementary Online Video). These findings suggest that the initial cardiac dysfunction of the donor heart was likely owing to TTS.

**Figure 2:**
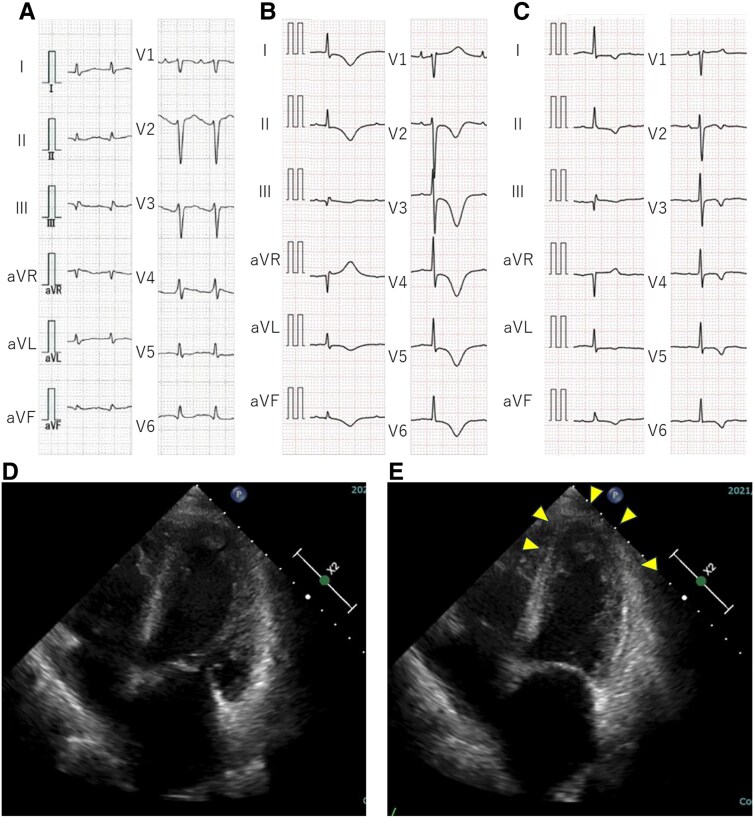
Electrocardiography and echocardiography during the second takotsubo syndrome episode. Electrocardiography on admission at another hospital (*A*), upon transfer to our hospital on day 5 (*B*), and at discharge (*C*). Echocardiography at the time of transfer to our hospital indicates apical hypokinesis (yellow arrowheads) at end-diastole (*D*) and end-systole (*E*).

Two years post-transplantation, the patient developed epilepsy secondary to a previous cerebral haemorrhage, requiring intubation on admission for restrictive thoracic disease from scoliosis. Despite preserved cardiac function (LV end-diastolic diameter, 38 mm; LVEF, 53%), she was discharged on ventilator support. Two years later (4 years post-transplantation), she was admitted to another hospital with difficulty breathing owing to phlegm-related airway obstruction. Upon arrival, her oxygen saturation was 92% with 10 L/min of supplemental oxygen, which improved to 100% following phlegm suctioning. Echocardiography indicated decreased biventricular apical wall motion, and ECG showed sinus tachycardia with flat T-waves (*Figure 2A*). She was transferred to our hospital 5 days post-admission. At transfer, her vital signs were stable, with no dyspnoea. ECG exhibited giant negative T-waves (*Figure 2B*), which improved at discharge (*Figure 2C*). Echocardiography at transfer highlighted hypokinesis of the apical biventricular wall (*Figures 2D* and *E*), which normalized within 10 days (see Supplementary Online Video). Coronary computed tomography and endomyocardial biopsy excluded coronary artery disease, myocarditis, and transplant rejection, leading to a TTS diagnosis.

**Figure 3:**
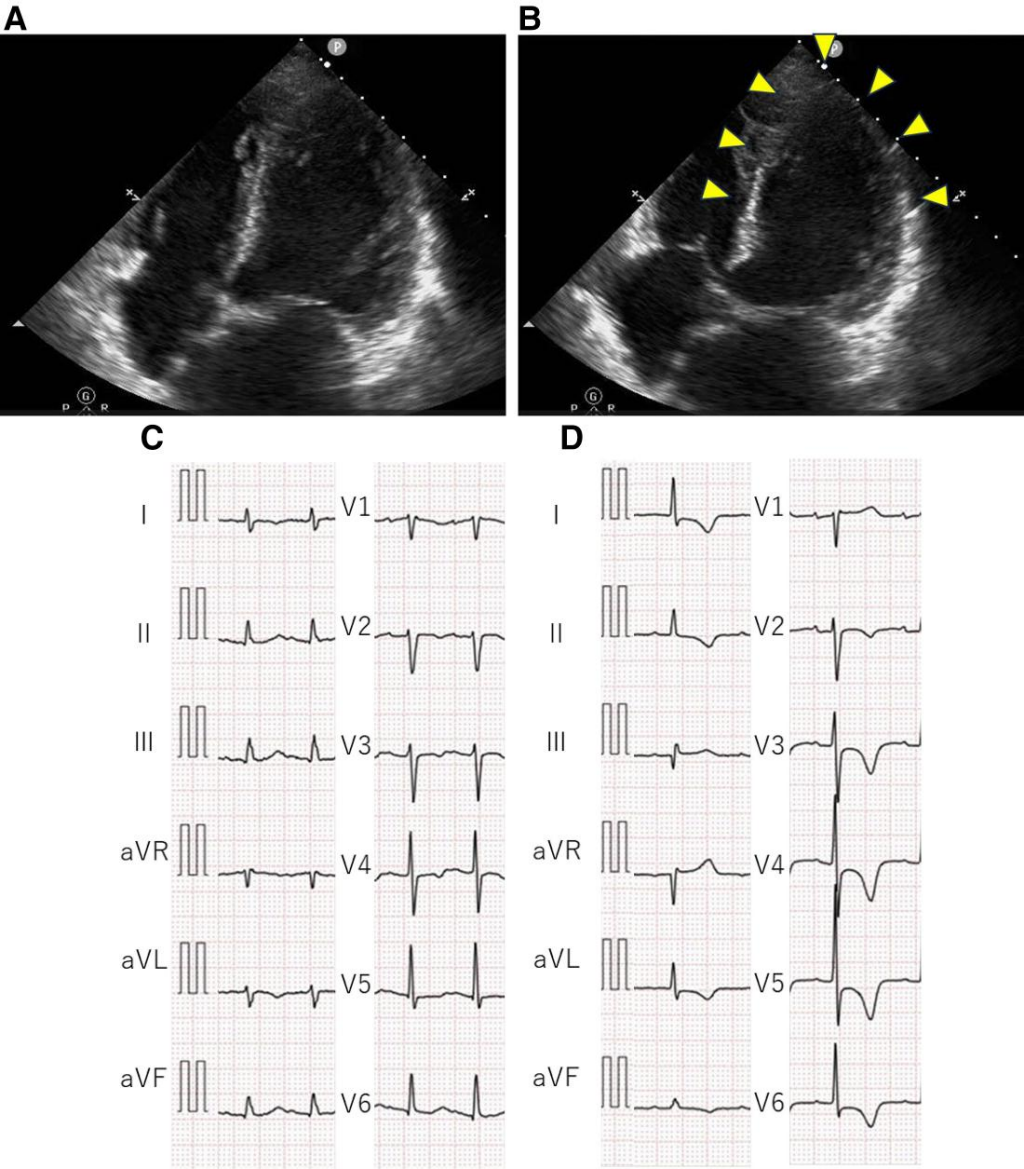
Electrocardiography and echocardiography findings during the third takotsubo syndrome episode. Echocardiography on admission demonstrates akinesis of the mid-to-apical biventricular wall (yellow arrowheads) at end-diastole (*A*) and end-systole (*B*). Electrocardiography on admission (*C*) and on day 6 (*D*).

**Figure 4:**
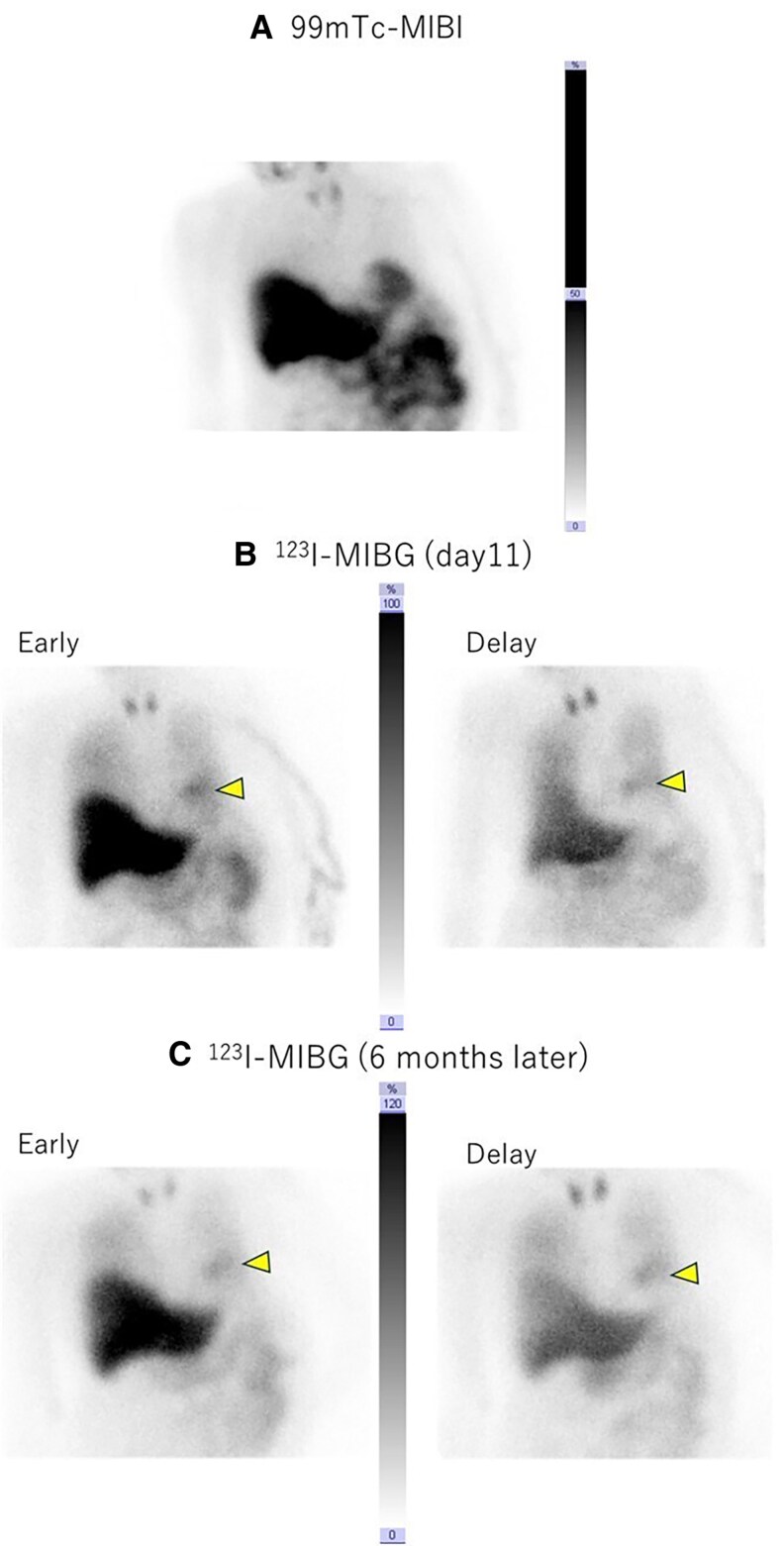
Cardiac scintigraphy images. Technetium-99 m sestamibi (99mTc-MIBI) scintigraphy performed following the third takotsubo syndrome episode shows preserved myocardial perfusion (*A*). The planar iodine-123 meta iodobenzylguanidine (^123^I-MIBG) images on day 11 during the third TTS episode demonstrate minimal myocardial uptake focused at the basal anterior wall (yellow arrowhead), and further decreased uptake particularly in the delayed phase compared with the early phase, indicating increased washout (*B*). ^123^I-MIBG scintigraphy taken 6 months later demonstrates no improvement in uptake, but the uptake is comparable between the early and late phase, indicating improvement in washout (*C*).

One-year later (5 years post-transplantation), the patient was readmitted to our hospital with breathing difficulty. Her oxygen saturation was maintained on 100% with 3 L/min of supplemental oxygen, and right heart catheterization revealed a low cardiac index (1.49 L/min/m^2^) and high pulmonary artery wedge pressure (19 mmHg). Low-dose dobutamine administration immediately improved symptoms. An echocardiogram showed an LV end-diastolic diameter of 41 mm and akinesis of the mid-to-apical biventricular wall (*Figure 3A* and *B*; see Supplementary Online Video). ECG showed sinus tachycardia and negative T-waves in leads V3 and V4 (*Figure 3C*). Coronary angiography and endomyocardial biopsy led to a diagnosis of recurrent TTS. On day 6 post-admission, biventricular wall motion was normalized (see Supplementary Online Video), and ECG showed typical giant negative T-waves as TTS (*Figure 3D*). Myocardial perfusion was preserved on technetium-99 m sestamibi scintigraphy (*Figure 4A*). Prior to discharge, the patient underwent ^123^I-MIBG scintigraphy on day-11, showing minimal myocardial uptake in the planar images, particularly focused at the basal anterior wall. Myocardial uptake in the delayed phase was further decreased compared with the early phase, indicating increased washout (*Figure 4B*). Carvedilol was initiated and titrated to 10 mg/day to mitigate exaggerated catecholamine responses. Repeat ^123^I-MIBG scintigraphy 6 months later showed no improvement in uptake, but uptake between the early and delayed phases was comparable, suggesting an improvement in the increased washout (*Figure 4C*). The patient remained stable without experiencing recurrence; however, at 7 years post-transplant (2 years after the previous episode), tracheostomy tube obstruction triggered electrocardiographic and echocardiographic changes resembling those of prior episodes, consistent with recurrent TTS.

## Discussion

We present a rare case of recurrent TTS in a heart transplant recipient, with a total of three occurrences pre- and post-transplantation. TTS is defined as a transient, reversible, systolic, ventricular dysfunction in the absence of coronary artery disease.^5^ While its precise mechanisms remain elusive, ^123^I-MIBG scintigraphy in acute phase TTS shows decreased uptake on delayed imaging and significantly increased washout, suggesting that increased cardiac sympathetic hyperactivity contributes to TTS development.^2,3^

A transplanted heart undergoes complete denervation at the time of transplantation. Bengal *et al*.^6^ evaluated post-transplantation sympathetic reinnervation using serial positron emission tomography and reported that sympathetic reinnervation occurred 18 months post-transplantation, starting from the basal anterior region then extending to the apical, septal, and lateral walls. The inferior wall remained denervated over the following 7 years, and the speed of reinnervation varied among individuals.^6^ In our patient, ^123^I-MIBG scintigraphy 5 years post-transplantation showed significantly decreased uptake, except for the basal anterior wall, suggesting insufficient reinnervation. This suggests that mechanisms unrelated to cardiac sympathetic hyperactivity may have been involved in TTS recurrence. Similar to our case, TTS was reported in a patient with insufficient sympathetic reinnervation post–heart transplantation,^7^ indicating that it may occur independently of cardiac sympathetic hyperactivity.

An increase in plasma catecholamine levels may be involved in TTS development. Plasma catecholamine levels are reported to be 2–3 times and 7–34 times higher in patients with TTS than in those with acute myocardial infarction and healthy individuals, respectively.^8^ In patients receiving a denervated transplanted heart, the absence of presynaptic neuronal uptake enhances inotropic sensitivity to circulating catecholamines, potentially contributing to TTS development.^9^ Furthermore, cardiac predisposition to TTS, passing from donor to recipient, may play a role in developing TTS. Our patient, with no TTS history despite multiple episodes of cerebral haemorrhages pre-transplantation, experienced two episodes post-transplantation from a donor with TTS. A similar case of TTS recurrence has been described in another transplant recipient from donor with TTS.^10^ Moreover, an *in vitro* induced pluripotent stem cell TTS model showed increased expression of cardiac stress markers and higher sensitivity to catecholamine.^11^

Given the critical nature of the patient’s LVAD complications and the need for heart transplantation, a marginal donor was accepted. LV systolic dysfunction occurs in up to 42% of patients with brain death.^12^ However, two large retrospective studies reported that the short- and long-term survival rates of recipients of donor hearts with initially low EFs do not differ significantly from those of donor hearts with normal EFs, and that the systolic function of the donor hearts improved post-transplantation.^13,14^ The International Society for Heart and Lung Transplantation guidelines recommend evaluation of reversible causes of dysfunction in donor hearts with initially low EFs, particularly in younger donors with severe brain injury history.^15^ Given severe donor heart shortages, transplantation of donor hearts with TTS is expected to become more common, underscoring the need for close post-transplantation monitoring.

Our case highlights the potential for developing recurrent TTS in transplant recipients of the hearts of donors with TTS, despite insufficient sympathetic innervation as evidenced using ^123^I-MIBG scintigraphy. Further research, including molecular and genetic analyses of susceptibility factors and histological analysis of endomyocardial biopsies during acute episodes, is warranted to elucidate the non-sympathetic-related mechanisms underlying TTS pathogenesis and development.

## Lead author biography


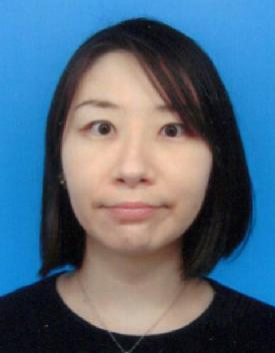


Kaori Yasumura is a cardiologist who has been involved in the management of patients with severe heart failure who require left ventricular assist devices or heart transplants at the University of Osaka Hospital.

## Supplementary Material

ytaf213_Supplementary_Data

## Data Availability

The data underlying this article are available in the article and in its online Supplementary material.

## Notes

**Consent:** The authors confirm that written consent for submission and publication of this case report including image(s) and associated text has been obtained from the patient in line with COPE guidance.

**Funding:** None declared.
